# Understanding the Dynamics: A Comprehensive Review of Family Therapy’s Impact on Expressed Emotions in Schizophrenia Patients

**DOI:** 10.7759/cureus.59491

**Published:** 2024-05-01

**Authors:** Anshita Girdhar, Ragini Patil, Apurva Bezalwar

**Affiliations:** 1 Psychiatry, Jawaharlal Nehru Medical College, Datta Meghe Institute of Higher Education & Research, Wardha, IND

**Keywords:** family dynamics, psychoeducation, treatment outcomes, psychiatry, expressed emotions, family therapy

## Abstract

This comprehensive review examines the impact of family therapy on expressed emotions (EE) within the context of psychiatric disorders. EE, characterized by high levels of criticism, hostility, or emotional over-involvement, have been consistently linked to poorer treatment outcomes and increased severity of psychiatric symptoms. The review explores various family therapy approaches and their effectiveness in reducing high EE levels in families of psychiatric patients. It synthesizes existing literature to highlight the mechanisms underlying the changes in EE, such as modifying communication patterns and enhancing family cohesion. Additionally, the review discusses the implications for clinical practice, emphasizing the importance of integrating family therapy into psychiatric treatment plans and providing psychoeducation to empower families to manage emotions effectively. Future research directions are also outlined, including investigating the long-term sustainability of changes brought about by family therapy and exploring cultural considerations in therapeutic approaches. Overall, the review underscores the pivotal role of family therapy in addressing EE and promoting recovery and resilience in psychiatric patients and their families.

## Introduction and background

Family therapy, also known as systemic therapy, is a therapeutic approach that focuses on understanding and addressing the dynamics within a family system to improve the mental health and well-being of its members. Unlike traditional individual therapy, family therapy views individuals within the context of their familial relationships and interactions. It seeks to identify and modify dysfunctional patterns of communication and behavior that contribute to psychological distress [1].

Expressed emotions (EE) refer to the level of emotionality expressed by family members toward a psychiatric patient, typically characterized by high levels of criticism, hostility, or emotional over-involvement. Research has consistently shown that high EE environments are associated with poorer treatment outcomes, higher rates of relapse, and increased severity of psychiatric symptoms across various disorders such as schizophrenia, bipolar disorder, and major depressive disorder [2].

The purpose of this review is to examine the impact of family therapy on EE in psychiatric patients. By synthesizing existing literature and empirical evidence, this review aims to explore the effectiveness of family therapy interventions in reducing high EE within families of psychiatric patients. Additionally, the review will investigate the mechanisms underlying the changes in EE brought about by family therapy, identify potential challenges and limitations, and discuss future directions for research and clinical practice in this area.

## Review

Understanding expressed emotions

Definition and Components of Expressed Emotions

**Figure 1:**
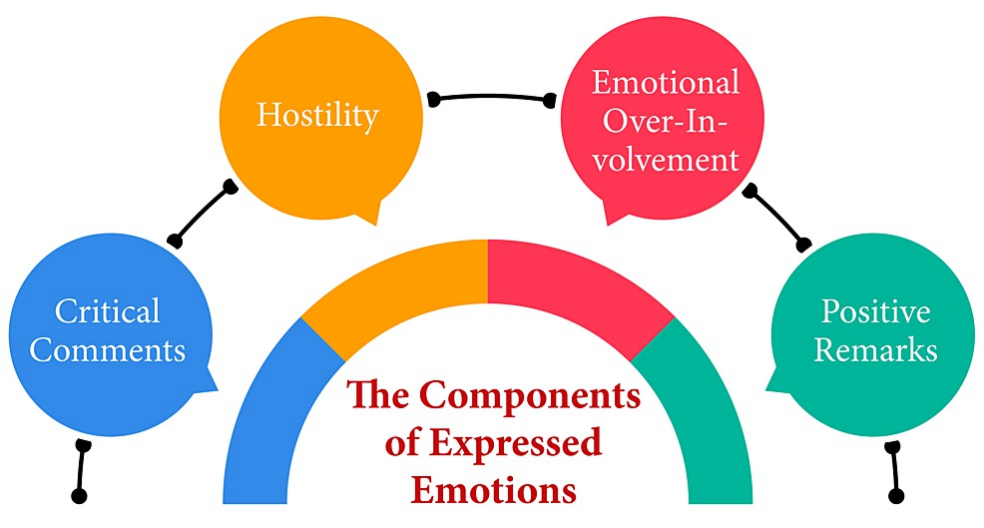
The components of EE The corresponding author self-created the image. EE, expressed emotions

EE serves as a metric of the familial atmosphere, gauging how family members spontaneously discuss a psychiatric patient. It specifically evaluates several facets of the family environment, encompassing critical remarks, hostility, and emotional over-involvement, sometimes incorporating positivity and warmth as indicators of a low-EE setting [3]. George Brown delineated five elements of EE: critical comments, hostility, emotional over-involvement, positive remarks (regard), and warmth [4]. In the context of schizophrenia, high EE, marked by critical comments, hostility, and emotional over-involvement, correlates with an elevated risk of relapse. At the same time, low EE entails warmth, empathy, and compassion toward the individual with schizophrenia [5,6]. The frequency with which these components are articulated determines whether the family environment is categorized as having high or low EE [5]. High EE has been associated with an increased probability of relapse in psychosis-related conditions, such as schizophrenia [5]. The components of expressed emotion are in Figure 1.

*Theoretical Frameworks Explaining Expressed Emotions* *in Psychiatric Patients*

Theoretical frameworks elucidating EE in psychiatric patients encompass a range of models and measures aimed at comprehending the influence of family dynamics on mental health conditions. George Brown's model, for instance, delineates five key components of EE: critical comments, hostility, emotional over-involvement, positive remarks (regard), and warmth [4]. High EE, characterized by critical comments, hostility, and emotional over-involvement, has been correlated with an elevated risk of relapse in individuals with mental illnesses such as schizophrenia and social anxiety disorder [2]. The stress-vulnerability model of psychosis posits that environments marked by high EE can serve as direct triggers for psychosis, thereby exacerbating mental illness [7]. Interventions rooted in family dynamics that mitigate high EE have effectively reduced patient relapse rates [4].

Common tools for assessing EE include the Camberwell Family Interview (CFI) and the Five-Minute Speech Sample (FMSS). The CFI is a semi-structured interview process that evaluates family members across various scales, including criticism, hostility, emotional over-involvement, warmth, and positive remarks [7,8]. Conversely, the FMSS involves soliciting relatives to speak freely about the patient for an uninterrupted five-minute duration, after which the content is analyzed for indicators of EE [9]. Theoretical frameworks explicating EE in psychiatric contexts underscore the significant role of family dynamics in shaping the trajectory of mental illness. Notably, interventions targeting high EE have demonstrated effectiveness in enhancing patient outcomes. The CFI and FMSS are commonly employed measures for assessing EE within research and clinical settings.

Measurement Methods for Assessing Expressed Emotions

Various methods are available for assessing EE in psychiatric patients, with the CFI and the FMSS standing out as the most widely utilized approaches [10-12]. The CFI involves a semi-structured interview process wherein family members are evaluated across five essential scales: criticism, hostility, emotional over-involvement, warmth, and positive remarks. In contrast, the FMSS entails asking relatives to speak candidly about the patient for five minutes, with subsequent analysis focusing on EE content [10-12]. Additionally, self-report measures have been developed to gauge EE, including the Level of Expressed Emotion Scale (LEE), the Expressed Emotion Adjective Checklist (EEAC), and the Questionnaire Assessment of Expressed Emotion (QAEE) [13]. The LEE comprises a 60-item questionnaire that assesses the perceived EE within the most significant relationships in the patient's life. Meanwhile, the EEAC consists of a 30-item checklist designed to evaluate the frequency of positive and negative comments made by family members regarding the patient. Finally, the QAEE is a 20-item questionnaire that measures the frequency and intensity of critical comments, hostility, and emotional over-involvement [13]. While the CFI and FMSS necessitate trained raters for EE assessment, a process that can be time-consuming and resource-intensive, self-report measures offer a more straightforward administration but may be susceptible to response biases. Nevertheless, regardless of the chosen method, it remains imperative to evaluate EE in psychiatric patients, given its established association with a heightened risk of relapse and poorer prognoses [4].

Family therapy approaches

Structural Family Therapy

Structural family therapy is a modality within family therapy that places a strong emphasis on the structural dynamics within the family unit, including its various subsystems. The central goal of this approach is to restructure the family system by carefully examining and adjusting the distribution of roles and power among family members. It emphasizes the importance of parental or adult caregiver authority and collaboration in establishing and enforcing appropriate boundaries for children. By fostering a cohesive and cooperative family dynamic, structural family therapy aims to enhance adult and sibling relationships while promoting healthier interactions [14,15]. Essential techniques employed in structural family therapy include mapping, visually representing and understanding the family structure, and identifying family interactions to discern the organization of the familial system. Central to this therapeutic model is the recognition and exploration of family structure and its constituent subsystems [16]. Developed by Salvador Minuchin, structural family therapy is a leading approach to systemic family intervention. It prioritizes establishing a well-defined hierarchical family structure, wherein distinct subsystems with clear boundaries and limits contribute to overall family functioning and cohesion [16].

Strategic Family Therapy

**Figure 2:**
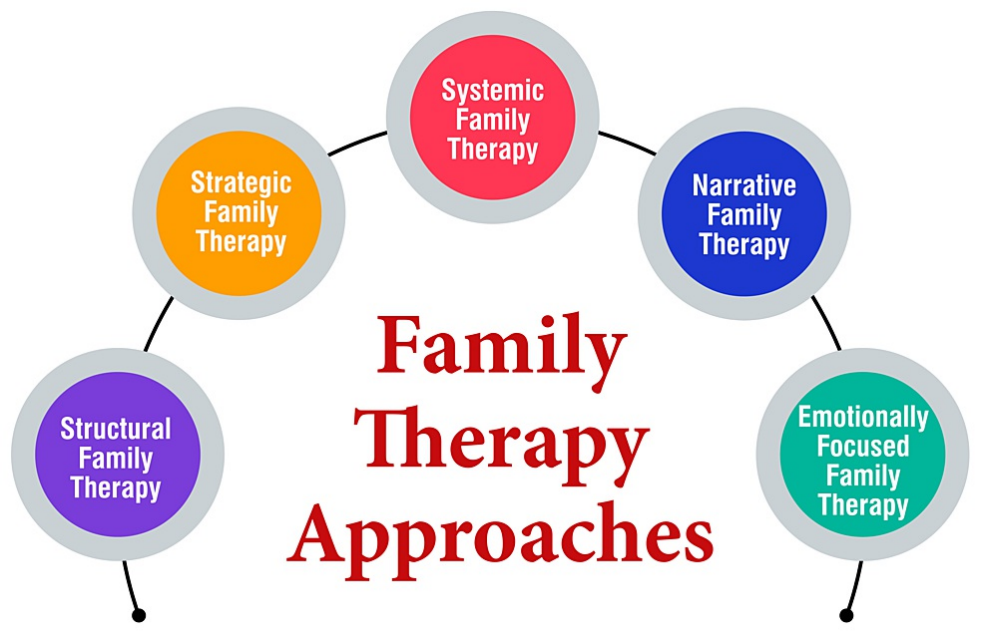
Family therapy approaches The corresponding author self-created the image.

Strategic family therapy represents a targeted approach, focusing on resolving specific issues within the familial structure, such as hierarchies, coalitions, and communication patterns [14]. Central to strategic family therapy is the notion of prescribing homework assignments designed to catalyze changes in the interactions among family members, particularly those involving the individual identified as bearing the "problem" or "symptom" [14]. Therapists within this framework diligently identify and address destructive patterns within the family system, replacing them with more constructive alternatives. The overarching objective is to forestall the emergence of further issues by fostering a supportive and nurturing familial environment [14]. Strategic therapy typically unfolds across five key stages: the social stage, problem stage, interactional stage, goal-setting stage, and task-setting stage [14]. Therapists guide families through a structured and sequential process toward identifying and implementing practical solutions to their challenges, promoting healthier relational dynamics, and improving overall family functioning. Family therapy approaches are shown in Figure 2.

Systemic Family Therapy

Systemic family therapy constitutes a form of psychotherapeutic intervention that places paramount importance on viewing the family as a cohesive unit, acknowledging the intricate interplay between symptomatology and the interpersonal milieu within which these symptoms manifest [17]. Rooted in the principles of family systems therapy, this approach posits that problems arise within a social and relational framework [18]. Rather than isolating individual conflicts, systemic family therapy endeavors to mitigate distress and discord by enhancing the overall systems of interaction within the family unit [18]. Essential strategies employed in systemic family therapy include deconstructing the presenting problem, identifying recurring patterns and feedback loops within family dynamics, addressing underlying beliefs and explanatory frameworks, attending to emotional dynamics and attachment patterns, and considering broader contextual factors [18].

The overarching objectives of systemic family therapy encompass enhancing communication channels, fostering a deeper understanding of and adeptness in managing unique family circumstances, cultivating a more harmonious and functional home environment, and harnessing the internal resilience and functional capacities inherent within the family structure [1,17]. Notably, this therapeutic approach proves particularly advantageous for families grappling with chronic neurotic conditions, pronounced EE, organic brain disorders, familial discord, and significant interpersonal conflicts [1]. Systemic family therapy strives to affect profound and enduring transformations in familial relationships and functioning through its comprehensive and holistic lens.

Narrative Family Therapy

Narrative family therapy represents a therapeutic modality centered on the narrative individuals construct and carry throughout their lifetimes. Its fundamental objective is assisting individuals in disentangling themselves from their problems and assuming authorship over their lives. By externalizing their issues, clients gain the capacity to perceive their challenges from diverse perspectives and uncover their life's purpose. Narrative family therapy proves especially advantageous for individuals and families grappling with the ramifications of various mental health conditions, including anxiety, ADHD, depression, attachment issues, eating disorders, grief, and PTSD. It distinguishes itself as a non-blaming, empowering, and collaborative approach, prioritizing recognizing individuals' skills and expertise in catalyzing transformative change in their lives [18-20]. In Narrative family therapy, therapists use techniques to facilitate clients' transcendence beyond distressing narratives. These may include positioning clients as observers within their own stories, fostering externalization of issues, and prompting consideration of alternative storylines and outcomes. Individuals can reimagine and reconstruct their narratives by engaging in this therapeutic process, cultivating healthier relationships and deeper self-understanding [19].

Emotionally Focused Family Therapy

Emotionally focused family therapy (EFFT) represents a form of family therapy designed to cultivate trust, mutual respect, and effective communication within familial relationships. Central to EFFT is fostering emotional security among all family members, enabling them to address issues and express concerns openly. By nurturing an environment where each member feels validated and heard, families can collaboratively engage in discussions, share feelings, and collectively seek solutions to challenges. EFFT therapists endeavor to establish a safe and accepting space wherein individuals can freely express themselves and have their perspectives understood and acknowledged [21,22]. As sessions progress, EFFT therapists facilitate discussions to unravel underlying emotions and identify patterns contributing to negative dynamics within the family. Through targeted interventions and guidance, families gradually develop enhanced communication skills, allowing each member to articulate their needs constructively. Moreover, as individuals learn to express themselves more openly, relationships within the family unit evolve and deepen. As a result of this therapeutic process, families often experience improved bonds and a sense of cohesion, facilitating the resolution of longstanding conflicts and the initiation of healing processes for past wounds [21,22].

Impact of family therapy on expressed emotions

Reduction of High Expressed Emotions​​​​​​​ in Family Members

Reducing high levels of EE among family members has demonstrated significant benefits in enhancing treatment outcomes and preventing relapses among individuals diagnosed with schizophrenia. Family therapy techniques, including psychoeducation, have proven effective in mitigating EE levels and fostering healthier familial interactions [23,24]. While existing research predominantly focuses on EE within families affected by schizophrenia, there is a growing acknowledgment of its relevance in other psychiatric conditions, such as social anxiety disorder and bipolar disorder [3]. Nevertheless, further investigation is warranted to comprehensively grasp the impact of EE reduction on treatment outcomes in these populations. Future research endeavors should continue exploring the efficacy of family therapy interventions in diminishing EE levels, thereby facilitating improved mental health outcomes for patients and their families [25].

Improvement in Communication Patterns

Family therapy has effectively restructured communication patterns and fostered improved family dynamics. Therapists play a pivotal role in guiding families to recognize and address unhealthy communication dynamics by facilitating open and empathetic dialogues, resolving past conflicts, and nurturing stronger, healthier bonds for the future [26]. Specific techniques employed in family therapy to enhance communication encompass active listening, assertiveness training, nonviolent communication, and conflict resolution skills [26,27]. Moreover, therapists offer real-time feedback during observation and application sessions, assisting in reshaping problematic patterns while also providing positive reinforcement to encourage constructive communication behaviors [28]. Counseling addressing family communication issues proves highly beneficial in aiding families in identifying and rectifying communication breakdowns, destructive behaviors, and recurring patterns, ultimately fostering overall well-being and family cohesion [29]. Often, family dynamics can be substantially improved through dedicated family therapy sessions, where families acquire and practice practical communication skills while resolving conflicts [30]. Through this collaborative therapeutic process, families enhance their communication abilities and cultivate a deeper understanding and appreciation for one another, promoting long-term harmony and unity within the family unit.

Enhancement of Family Cohesion and Support

The notion of family cohesion is crucial in bolstering the well-being of individuals within a family, particularly concerning mental health. The research underscores that dysfunctional levels of family cohesion correlate with mental health challenges, emphasizing the potential benefits of fostering enhanced family cohesion for both patients and their families [31,32]. Employing strategies to fortify family communication, such as active listening, forgiveness, and boundary-setting, can play a pivotal role in ameliorating family dynamics and cultivating a nurturing emotional atmosphere [28,30]. In mental health, family therapy enhances family dynamics and communication. Family therapists are adept at equipping families with practical communication skills, facilitating conflict resolution, and fostering expressions of affection, all of which contribute to a healthier family dynamic [30]. Furthermore, family therapy serves as a conduit for addressing problematic patterns and extending support to individuals grappling with mental health challenges, thus fostering an overarching enhancement in family cohesion and support [28]. Through collaborative therapeutic interventions, families can develop stronger bonds, navigate challenges more effectively, and provide a robust support network for each member, thereby fostering resilience and well-being amidst mental health struggles.

Empirical Evidence Supporting the Effectiveness of Family Therapy in Modifying Expressed Emotions

The influence of family therapy on altering EE in psychiatric patients, particularly individuals with schizophrenia, has garnered significant research attention. EE, encompassing family members' attitudes toward a patient, serves as a pivotal predictor of treatment outcomes and relapse rates in psychiatric disorders, notably schizophrenia [24]. Family-focused interventions, such as family psychoeducation, have been developed to reshape family environments and mitigate the adverse effects of EE. These interventions have exhibited promise in reducing elevated EE levels and forestalling relapses among individuals with schizophrenia [33]. While empirical evidence supports the efficacy of family therapy in modifying EE, it is pertinent to acknowledge that its impact on EE within other psychiatric populations, such as those with a social anxiety disorder or bipolar disorder, remains less established [34]. Existing literature suggests that family therapy, particularly family-based interventions, holds the potential for modifying EE in psychiatric patients, particularly those diagnosed with schizophrenia. Nonetheless, further research endeavors are warranted to elucidate its impact across diverse psychiatric diagnoses comprehensively. By expanding our understanding of the efficacy of family therapy in altering EE, we can refine and tailor interventions to meet the unique needs of individuals across various psychiatric conditions, thereby optimizing treatment outcomes and fostering improved quality of life.

Mechanisms of change

Role of Therapist in Modifying Expressed Emotions

The role of therapists in modifying EE within the framework of family therapy and its impact on psychiatric patients represents a significant area of investigation. Mechanisms of change in psychotherapy, including family therapy, encompass the processes by which therapeutic interventions lead to symptom alleviation and broader changes in clients extending beyond the therapy setting [35,36]. These mechanisms may entail factors such as the therapeutic alliance, interpretations of transference, attainment of insight, and specific therapeutic processes occurring within sessions [37]. In family therapy, the therapist's role in modifying EE involves facilitating shifts in the attitudes and interactions of family members toward the patient, fostering adaptive perspectives and behaviors, and restructuring relationships within the family system [36]. Furthermore, concerning psychiatric patients, therapists endeavor to reduce elevated EE levels, such as critical or hostile comments, through interventions to bolster social support, mitigate interpersonal stress, facilitate emotional expression, and enhance interpersonal skills within the familial milieu [38]. Overall, therapists serve as instrumental agents in identifying and targeting specific mechanisms of change within the family system to modify EE, thereby fostering improved outcomes for psychiatric patients. By adeptly navigating and intervening within the family dynamics, therapists facilitate transformative shifts that contribute to the overall well-being and resilience of individuals grappling with psychiatric challenges.

Family Dynamics and Their Influence on Expressed Emotions

The mechanisms of change within family dynamics and their impact on EE in the context of psychiatric patients are intricate and multifaceted. Extensive research underscores the significance of comprehending the causal interplay between work and family constructs, alongside the repercussions of family dynamics on EE and youth psychopathology. Family systems theory and work-family research illuminate the interconnectedness of work and family dynamics, stressing the imperative of discerning causal relationships among various constructs within the family system [39]. Moreover, parental EE, gauging how families respond to a member's psychiatric disorder, has been linked to youth psychopathology, with emotionally charged family dynamics potentially exacerbating psychiatric conditions and complicating recovery processes [40]. Furthermore, existing literature highlights the necessity for further investigation to elucidate the specific mechanisms through which family dynamics influence EE and psychopathology. This entails exploring attributions of causality, interaction patterns, and bidirectional-interactional models of toxic family stress and formulating mechanistic models to inform treatment and intervention strategies [40]. The intricate causal relationships within the family system underpinning mechanisms of change in family dynamics and their influence on EE bear significant implications for the development and management of psychiatric disorders in both adults and youth. Ongoing research endeavors are crucial for unraveling these mechanisms comprehensively and harnessing their potential to inform efficacious interventions aimed at improving outcomes for individuals grappling with psychiatric challenges.

Psychoeducation and Skill-Building in Family Therapy

**Figure 3:**
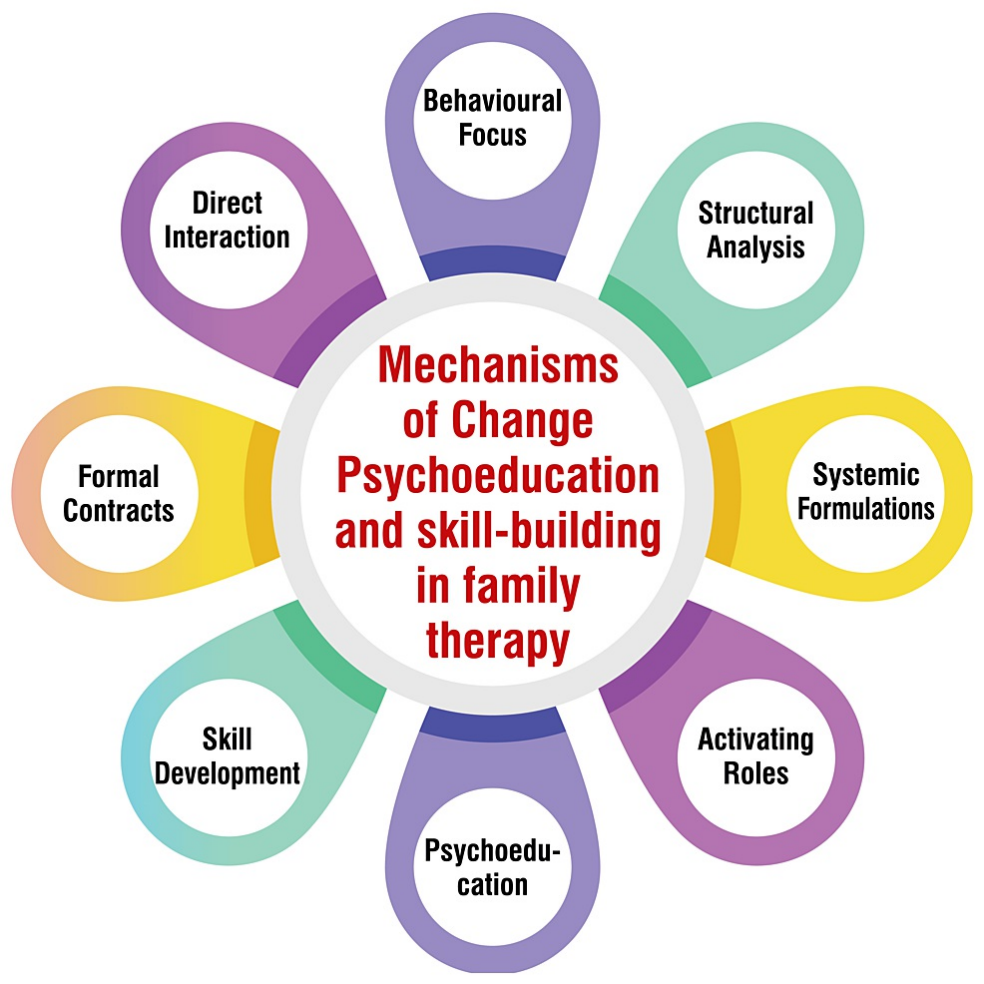
Mechanisms of change: psychoeducation and skill-building in family therapy The corresponding author self-created the image.

The mechanisms of change in family therapy, mainly through psychoeducation and skill-building, encompass several fundamental principles and techniques. Family therapy, as a structured form of psychotherapy, aims to alleviate distress and conflict by enhancing family systems and relationships. Unlike traditional individual therapy, family therapists focus on the interactions between family members rather than solely on individual psychology. The change process in family therapy often fosters new relational experiences among family members, emphasizing the present moment and addressing conflicts concretely and abstractly that cannot be together fundamentally. Family therapists assume various roles, including activator, challenger, supporter, interpreter, re-integrator, and educator, to facilitate change within the family system [1]. These roles enable therapists to encourage introspection, challenge unproductive patterns, provide emotional support, interpret underlying dynamics, facilitate reconciliation, and impart knowledge and skills. Family-based interventions, such as psychoeducation, have demonstrated the potential to reduce high EE levels and prevent symptom relapse in patients with schizophrenia [24]. While the specific mechanisms of change through psychoeducation and skill-building in family therapy may not be explicitly outlined in all sources, the overarching principles and techniques of family therapy suggest that these mechanisms involve creating new relational experiences, enhancing communication, addressing maladaptive interactional family patterns, and mobilizing the family's inherent strengths and resources [1]. By empowering families with knowledge, skills, and a supportive environment, family therapy promotes lasting change and resilience within the familial context. Mechanisms of change are shown in Figure 3.

Challenges and limitations

Reluctance of Families to Engage in Therapy

One of the significant challenges and limitations of family therapy lies in the reluctance of families to engage in the therapeutic process. Several factors contribute to this reluctance, including issues such as poor boundaries within the family, feelings of resentment toward specific family members, fear of judgment or targeting, and the perception that family therapy is time-consuming and arduous [41]. Moreover, in some instances, an individual family member grappling with their mental health struggles may find it challenging to actively participate and engage emotionally and cognitively in family therapy sessions, thereby impeding progress [41,42]. Additionally, families may hesitate to address their mental health concerns openly in sessions due to the stigma associated with mental illness or involvement within the criminal justice system [42].

Family therapists are urged to emphasize the added value that comes from families receiving treatment collectively and to remain cognizant of how the stigma surrounding mental health and involvement within the criminal justice system may influence family members' willingness to disclose and address their mental health concerns [42]. Therapists must gain insight into the unique stressors experienced by each family and adopt a strength-based approach that harnesses family members' competencies and motivations, thereby fostering optimal therapeutic outcomes [43]. By recognizing and addressing these challenges with sensitivity and understanding, therapists can cultivate an environment conducive to meaningful engagement and progress within family therapy sessions.

Cultural Considerations in Family Therapy and Expressed Emotions

Cultural considerations in the context of family therapy and EE can indeed present numerous challenges and limitations. Within families, difficulties such as poor boundaries, resentment toward specific members, or a family member struggling with mental health issues, such as PTSD, can hinder the effectiveness of family therapy [41]. Additionally, cultural factors, including stigma surrounding mental health and the bidirectional relationship between mental health and family dynamics, can significantly influence both the efficacy of family therapy and the expression of EE [42]. Moreover, the absence of clear protocols for identifying families in need of therapy, coupled with the scarcity of therapeutic resources and the substantial emotional toll on caregivers, further complicate the implementation of family therapy [44]. Therefore, the role of family therapy in addressing cultural considerations and its impact on EE represent crucial areas warranting further research. A deeper understanding of how cultural factors shape family dynamics, attitudes toward mental illness, and help-seeking behaviors can enhance the effectiveness of family therapy in managing EE within diverse cultural contexts. Additionally, developing culturally sensitive approaches and clear protocols for identifying families in need of therapy can help alleviate some of the challenges and limitations associated with cultural considerations in family therapy and EE [45]. By integrating cultural competence into therapeutic practices and tailoring interventions to meet the unique needs of diverse populations, family therapists can foster greater engagement and positive outcomes within multicultural settings.

Long-Term Sustainability of Changes in Expressed Emotions

The long-term sustainability of changes in EE within the context of psychiatric patients, particularly individuals with schizophrenia, presents several challenges and limitations. Extensive research consistently demonstrates that elevated levels of EE within the family environment correlate with poorer clinical outcomes, reduced treatment responsiveness, and heightened rates of relapse across various psychiatric disorders, including schizophrenia, major depressive disorder, and bipolar disorder [3,40]. Family-based interventions, such as family psychoeducation, show promise in mitigating high EE levels and preventing symptom relapse in patients with schizophrenia [3]. However, the widespread implementation of such interventions encounters obstacles, including the limited uptake of the EE construct in community mental health settings and the necessity for strategies to address organizational implementation barriers [3,46].

Furthermore, evidence suggests that the sustainability of transitioning from high EE to low EE is achievable when interventions are delivered in a group therapy format [3]. Nonetheless, a paradigm shift in clinical interventions is imperative, necessitating a departure from deficit models toward resilience or strength-based approaches [3]. By emphasizing individuals' inherent strengths and building upon familial resources, clinicians can foster lasting changes in EE dynamics, promoting sustained improvements in mental health outcomes. Efforts to address these challenges and embrace resilience-focused interventions hold promise for enhancing the long-term sustainability of changes in EE and fostering greater resilience among psychiatric patients and their families.

Integration of Family Therapy With Other Psychiatric Treatments

Integrating family therapy with other psychiatric treatments has demonstrated effectiveness in improving outcomes for individuals grappling with mental illness and addiction. The research underscores that behavioral health treatment incorporating family therapy yields superior results compared to treatments lacking such integration. For individuals contending with mental illness, combining family therapy with individual treatment enhances medication adherence, reduces rates of relapse and rehospitalization, alleviates psychiatric symptoms, and mitigates stress. Similarly, for individuals struggling with addiction, family therapy aids in decision-making regarding treatment initiation or continuation and diminishes the likelihood of treatment dropout [47]. However, there exist challenges and limitations when integrating family therapy with other psychiatric treatments. These include the imperative for active participation from all family members, the substantial time commitment required, the potential lack of individual focus within group sessions, and the risk of exacerbating family discord if therapy is not conducted adeptly [41,48]. Addressing these challenges necessitates ensuring the commitment of all family members to the therapeutic process, providing individual therapy when indicated, and engaging a seasoned therapist capable of skillfully mediating family sessions. Despite these obstacles, the documented benefits of integrating family therapy with other psychiatric treatments are substantial, significantly contributing to the holistic well-being of individuals and their families [42]. By capitalizing on the synergistic effects of family therapy and other treatment modalities, clinicians can enhance treatment outcomes and foster resilience within familial networks, ultimately promoting sustained recovery and improved quality of life for individuals grappling with mental illness and addiction.

## Conclusions

In conclusion, this review highlights the significant impact of family therapy on addressing EE within families of psychiatric patients. Through exploring various therapeutic approaches, it becomes evident that family therapy interventions effectively reduce high EE, thereby improving treatment outcomes and enhancing family functioning. By targeting dysfunctional communication patterns, fostering cohesion, and providing psychoeducation, family therapy equips families with the necessary tools to navigate emotional challenges more effectively. These findings underscore the importance of integrating family therapy into psychiatric treatment plans, emphasizing the need for clinicians to prioritize family involvement and support. Moving forward, future research should focus on investigating the long-term sustainability of changes brought about by family therapy, as well as exploring cultural considerations and the integration of family therapy with other psychiatric interventions. Ultimately, family therapy stands as a pivotal tool in promoting recovery and resilience in psychiatric patients by addressing the systemic dynamics that contribute to emotional distress within the family unit.

## Figures and Tables

**Figure 4:**
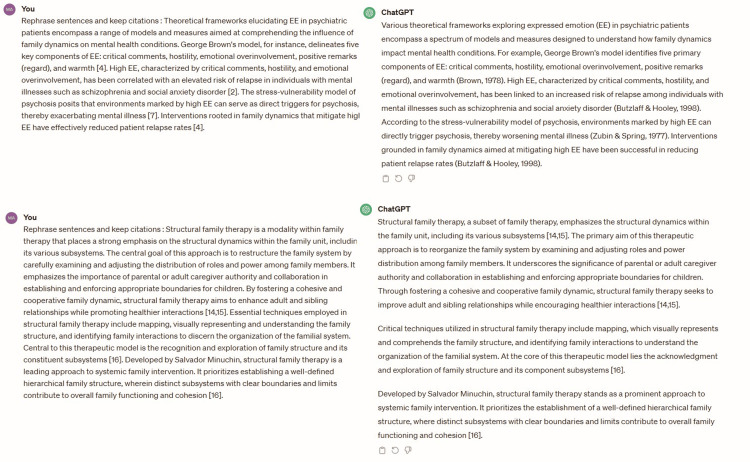
ChatGPT-assisted editing and rephrasing of the review article

## Appendices

The ChatGPT language model (OpenAI, San Francisco, California) was employed to assist in formulating key arguments, structuring the content, and refining the language of our manuscript. It provided valuable insights and suggestions throughout the writing process, enhancing the overall coherence and clarity of the article. It was also utilized to assist in editing and rephrasing the work to ensure coherence and clarity in conveying the findings (Figure 4).
